# A Stereotaxic Atlas of Primary Cortical Areas in the Developing Rat Brain from Postnatal Days 8 to 20

**DOI:** 10.1523/ENEURO.0237-26.2026

**Published:** 2026-11-03

**Authors:** Nicholas J. Sattler, Anna K. Grobengieser, James C. Dooley

**Affiliations:** ^1^Department of Biological Sciences, Purdue University, West Lafayette, Indiana 47906; ^2^Purdue Institute for Integrative Neuroscience, Purdue University, West Lafayette, Indiana 47906

**Keywords:** cortical development, neurodevelopment, primary auditory cortex, primary somatosensory cortex, primary visual cortex

## Abstract

Postnatal brain growth is nonlinear, making precise stereotaxic targeting in the developing rat neocortex difficult without age-specific knowledge of cortical area locations. Traditional atlases visualize brain slices in the coronal plane, which can obscure top-down areal boundaries and subdomains. To address these shortcomings, we created a developmental stereotaxic atlas that maps the neocortex of postnatal day (P) 8, P12, P16, and P20 in male and female Sprague Dawley and Long–Evans rats onto a coordinate grid. After using a stereotaxic device to create a grid of fluorescent probes, we extracted the brain, flattened neocortical tissue, and stained it for cytochrome oxidase, which is predominantly found in layer IV of primary cortical areas. Reconstructions of the primary somatosensory, auditory, and visual cortices demonstrate high structural reproducibility within each age and strain group. Our atlas captures the location of primary cortical areas at these four ages, showing that neocortical expansion is nonisometric, expanding preferentially along the rostral-caudal axis. Finally, we complement these top-down maps by extracting local neocortical surface angles from an existing coronal atlas, enabling proper electrode orientation to be perpendicular to the developing neocortex. Ultimately, this anatomically verified resource provides a standardized blueprint that eliminates resource-intensive trial-and-error mapping and maximizes experimental reproducibility in developmental systems neuroscience.

## Significance Statement

Targeting specific neocortical areas in developing rats is uniquely challenging because nonlinear brain expansion renders scaled adult coordinates inaccurate, while traditional coronal sections obscure top-down areal boundaries. To resolve this, we established a top-down stereotaxic atlas that maps primary sensory cortices onto flattened, cytochrome oxidase-stained tissue across early postnatal development (P8–P20) in both Sprague Dawley and Long–Evans rats. By combining equidistant coordinate grids with empirical cortical surface angles, this resource provides an accurate, reproducible surgical blueprint. This reference tool eliminates trial-and-error coordinate mapping, reduces animal waste, and maximizes experimental precision for the developmental neuroscience community.

## Introduction

The rat has long been one of the most popular experimental organisms used in behavioral neuroscience, with the first comprehensive adult rat brain atlas published over 60 years ago (König and Klippel, 1963). These tools, along with stereotaxic devices, have enabled researchers to accurately target pharmacological injections and neurophysiological recordings in specific cortical areas and subcortical nuclei of the adult rat brain. More recently, methodological innovations have pushed the boundaries of developmental neuroscience, making neurophysiological recordings and stereotaxic injections in the neocortex of developing rats increasingly common (Dooley and van der Heijden, 2024). This technological shift has driven the need for atlases in developing rats like those found in adults. Because brain development is highly nonlinear—particularly in the neocortex—simply “scaling down” adult coordinates results in significant stereotaxic errors. Consequently, this has led individual labs to determine their own coordinates, resulting in unnecessary duplication that wastes both time and animal life.

To answer this need, atlases of the developing rat brain have been published for embryonic (Altman and Bayer, 1995) and postnatal ages (Zilles, 2012; Khazipov et al., 2015; Ramachandra and Subramanian, 2016; Chen et al., 2022; Bajic et al., 2023), some of which incorporate stereotaxic coordinates and histological staining. However, identifying and aiming for precise subregions within primary cortical areas is constrained by the traditional coronal perspective of existing atlases and a lack of distinctive structural markers in the developing neocortex. Without a top-down, overhead view of areal boundaries, navigating neocortical areas can remain challenging.

Here, we present a developmental neocortical atlas that maps stereotaxic coordinates directly onto flattened tissue stained for cytochrome oxidase (CO). CO readily labels layer IV of primary sensory areas from postnatal day (P) 5 through adulthood (Seelke et al., 2012). Reconstructions of stereotaxic coordinates on top of CO-stained tissue are presented for rats at P8, P12, P16, and P20. These coordinates have been successfully used in several previous studies by our laboratory (Dooley and Blumberg, 2018; Reid et al., 2026) and are consistent with published values used by other labs that study the development of the rat neocortex.

## Materials and Methods

A total of eight male and female Sprague Dawley (6 M, 2 F) and eight male and female Long–Evans (3 M, 5 F) Norway rats (*Rattus norvegicus*) were used from P8 to P20, which included two rats per age. Breeding stock was acquired from commercial vendors or bred in-house from existing colonies. When commercial vendors were used, Long–Evans breeders were obtained from Envigo (now Inotiv) and Sprague Dawley breeders from Charles River Laboratories. All experimental animals were conceived, born, and reared in-house in their respective breeding colonies under identical institutional housing conditions. For each age and strain, the animal with the highest histological quality was selected for inclusion in the atlas. Histological quality was operationally defined by two criteria: (1) optimal tangential sectioning, such that layer IV across primary sensory areas was captured across the minimum number of sequential sections (typically 2–3), and (2) high contrast and clear laminar/areal differentiation of CO staining, allowing unambiguous identification of primary sensory borders and individual barrel walls. Pup sex, weights, and bregma–lambda distances can be seen in Table 1 and Figure 1.

**Figure 1.**
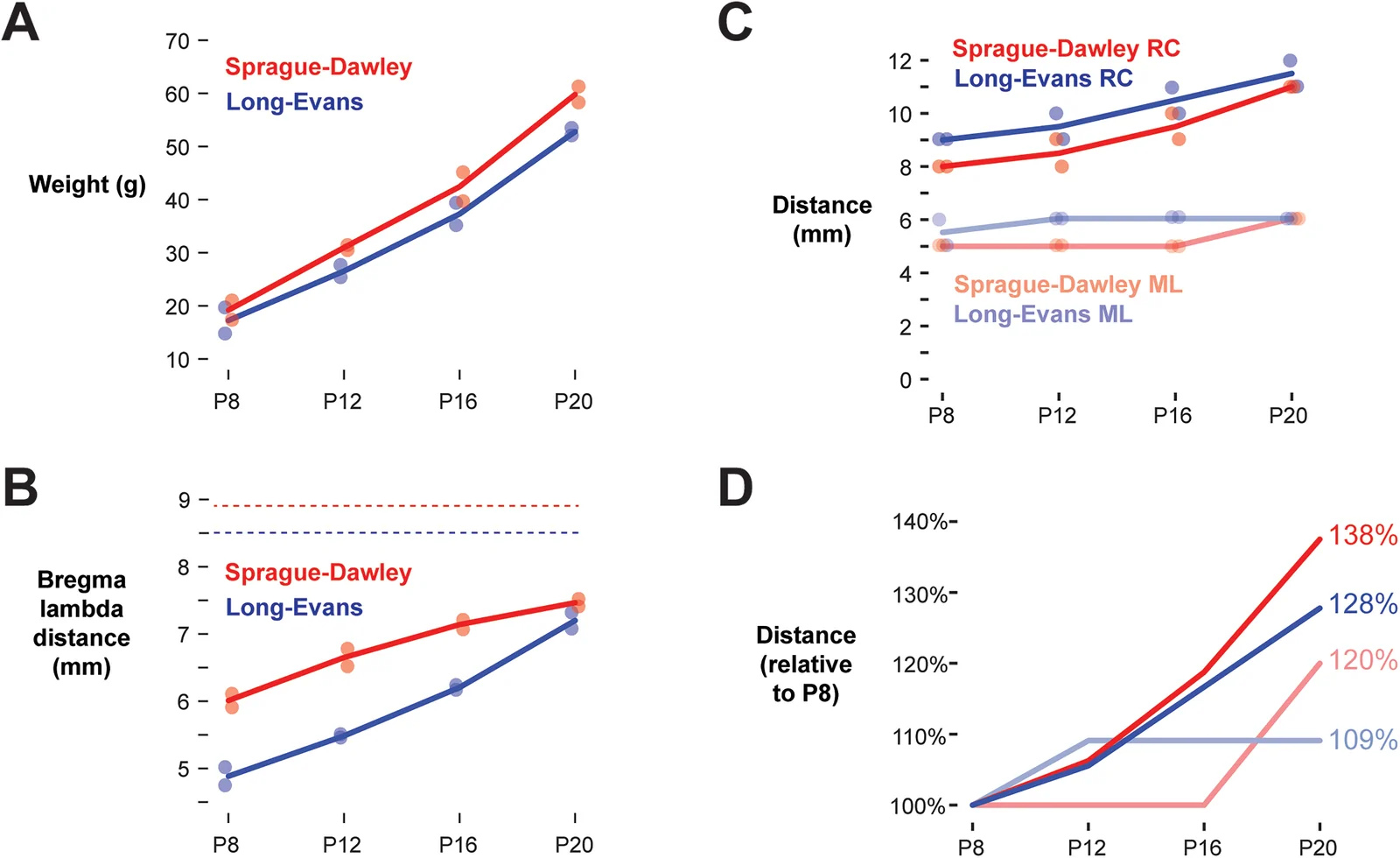
Postnatal body and skull growth across age by strain. ***A***, Total body weight (g) across early development. Colored dots indicate individual subject weights, and solid lines track the group mean. ***B***, Bregma–lambda distances (mm) across development. Solid lines connect early postnatal group means. Horizontal dotted lines at the top of the plot represent reference adult bregma–lambda distances for each strain (red, Sprague Dawley; blue, Long–Evans), illustrating that cranial growth has not yet reached adult levels at P20. ***C***, Neocortical map extent (mm) measured along the rostral-caudal (RC; dark lines) and medial-lateral (ML; light lines) axes from P8 to P20 for Sprague Dawley (red) and Long–Evans (blue) rats. Individual subject points and mean lines are shown. ***D***, Relative neocortical expansion normalized to P8 dimensions, demonstrating that rostral-caudal elongation exceeds medial-lateral widening in both strains.

**Table 1.** Bregma–lambda distance, weights, and sex for all included animals

*Age*	*B*–*L distance (mm)*	*Weight (g)*	*Sex*
	*Long–Evans*		
*P8*	*5.02*	*19.7*	*F*
	*4.75*	*14.8*	*M*
*P12*	*5.51*	*27.7*	*M*
	*5.46*	*25.4*	*F*
*P16*	*6.17*	*35.2*	*F*
	*6.24*	*39.4*	*F*
*P20*	*7.32*	*52.1*	*M*
	*7.08*	*53.5*	*F*
	*Sprague Dawley*		
*P8*	*5.91*	*21.02*	*M*
	*6.11*	*17.39*	*F*
*P12*	*6.78*	*31.45*	*M*
	*6.52*	*30.5*	*F*
*P16*	*7.21*	*45.16*	*M*
	*7.07*	*39.7*	*M*
*P20*	*7.52*	*61.3*	*M*
	*7.41*	*58.3*	*M*

Pups were born to mothers housed in laboratory cages (48 × 20 × 26 cm) with a 12 h light/dark schedule. Food and water were freely available. Expecting mothers were checked for pups daily, and the day of birth was considered P0. If litters consisted of more than eight pups, litters were culled down to eight pups (with equal numbers of males and females if possible) on or before P3 to ensure similar maternal care and reduce variability. The two animals at each age and strain always came from different litters. All experiments were conducted in accordance with the National Institutes of Health (NIH) Guide for the Care and Use of Laboratory Animals (NIH Publication No. 80–23) and were approved by the Institutional Animal Care and Use Committees of the University of Iowa and Purdue University.

### Surgery

Pups with a visible milk band were removed from the litter and put under isoflurane anesthesia (3–5%; Phoenix Pharmaceuticals). For P16 and P20 pups, the scalp was shaved, and ophthalmic ointment was applied to the eyes. Pups were injected with the anti-inflammatory drug carprofen (5 mg/kg, s.c.; Putney), and an incision was made down the midline of the scalp. Pups were secured in a stereotaxic apparatus (Stoelting) using nonpuncture ear bars and a nose cone (for P8 and P12 pups) or an incisor bar (for P16 and P20 pups). Standardized head leveling was confirmed in both planes using a stereotaxic microprobe: rostral-caudal tilt was adjusted until the dorsal-ventral (DV) height difference between bregma and lambda was within 0.05 mm, and medial-lateral level was verified by ensuring that DV coordinates measured at symmetric positions roughly midway between bregma and lambda (±2.0 mm lateral from the sagittal suture) also differed by <0.05 mm. Following leveling, the entire left side of the skull above the neocortex was carefully removed using dura scissors. The brain was flushed as needed with sterile saline to prevent desiccation. A 32-gauge needle was coated with fluorescent DiI (Vybrant DiI Cell-Labeling Solution; Life Technologies) and attached to a stereotaxic arm (Fig. 2*A*). Bregma was defined as the origin of a grid of points on the neocortex, and the needle was inserted into the cortex to a depth of at least 2 mm in a 1 mm grid, including as many locations in the neocortex as the craniotomy allowed (Fig. 2*B*). The needle was reinserted into DiI after three insertions to ensure it remained coated in fluorescent dye.

**Figure 2.**
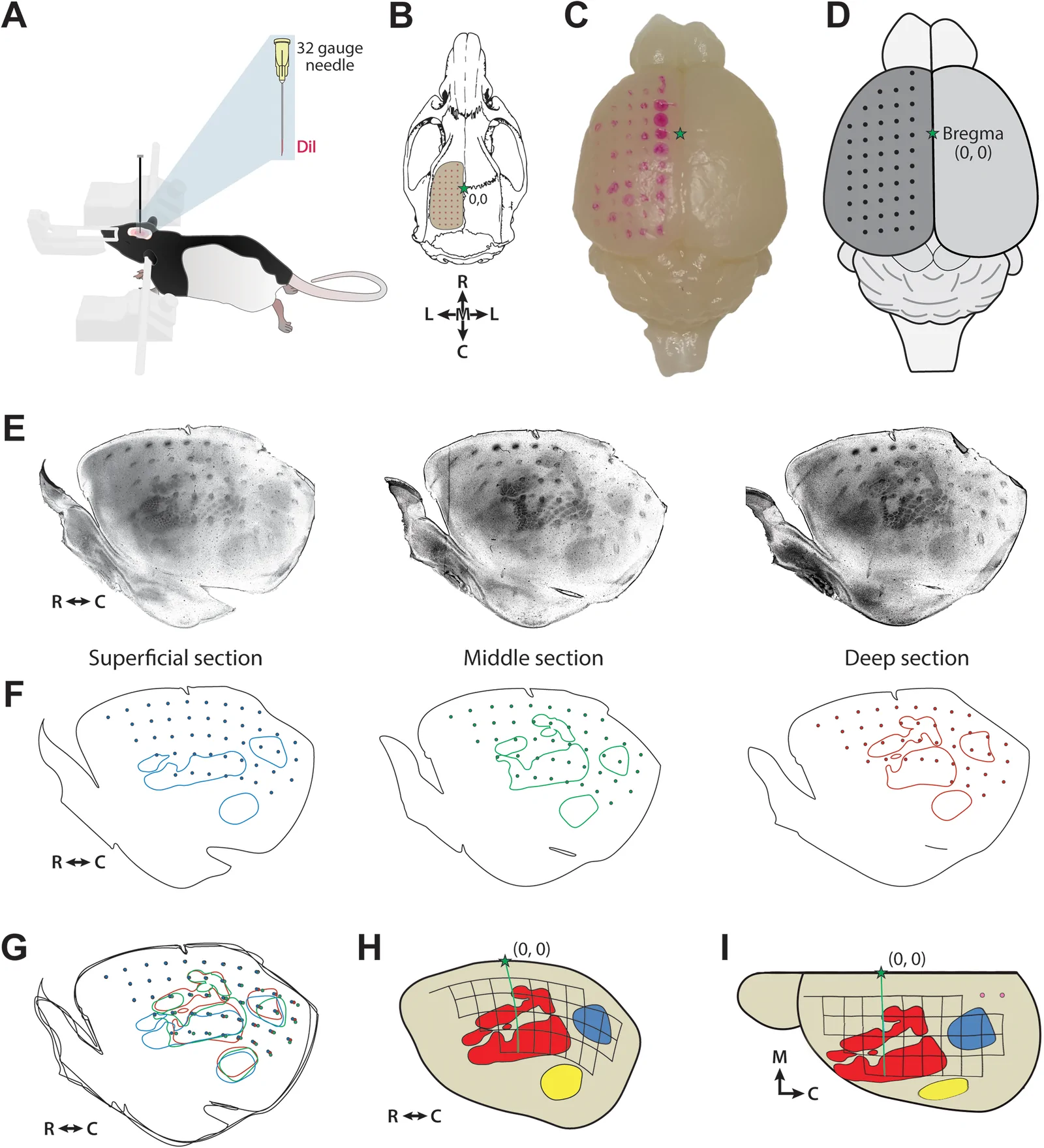
Methodology for stereotaxic coordinate mapping and top-down neocortical reconstruction. ***A***, Schematic of a rat pup secured in a stereotaxic frame. Inset shows a 32-gauge needle coated with the fluorescent DiI, which is inserted into the neocortex in a 1 mm grid pattern. ***B***, Dorsal schematic of the skull with a craniotomy exposing the left cortical hemisphere, showing the stereotaxic grid pattern relative to bregma (0, 0; green star). R, rostral; C, caudal; M, medial; L, lateral. ***C***, Dorsal view photograph of an extracted, postfixed whole brain displaying the physical grid layout of the fluorescent DiI markers on the surface of the neocortex. ***D***, Illustration of the brain in ***C***, showing the surface coordinate points relative to bregma (0, 0). ***E***, Serial tangential sections of the flattened cortical hemisphere stained for CO. Rostral is to the left, medial is to the top. Panels represent superficial, middle, and deep sections of a representative P12 brain, revealing distinct layer IV boundaries and DiI tissue marks. ***F***, Corresponding line tracings of the serial sections in ***E***, isolating the individual probe locations (dots) and anatomical boundaries (lines) unique to the superficial (blue), middle (green), and deep (red) tissue planes. ***G***, Composite overlay of three serial sections in ***F***, combining all sections into a single comprehensive reconstruction. ***H***, Top-down reconstruction of the flattened hemisphere, aligning the corrected coordinate grid with defined primary sensory fields (S1, red; V1, blue; A1, yellow) relative to bregma. ***I***, Final straightened stereotaxic grid template (when viewed from directly overhead) showing coordinate references mapped directly onto the boundaries of the primary cortical areas. Pink-filled dots indicate grid coordinates not seen in the reconstruction.

Following complete mapping of the exposed neocortex, the pup was killed with an overdose of 10:1 ketamine/xylazine (Covetrus; >100 mg/kg) and perfused with phosphate-buffered saline (PBS; Sigma-Aldrich), followed by 4% paraformaldehyde (Sigma-Aldrich). The brain was immediately extracted (Fig. 2*C*,*D*) and postfixed in 4% paraformaldehyde for at least 24 h. At least 24 h before brain sectioning, it was transferred to a 20% solution of sucrose (Sigma-Aldrich) in PBS (PB-sucrose) until it was no longer buoyant in solution.

### Histology

The cortical hemispheres were dissected apart from the underlying tissue, including the hippocampus and basal ganglia, and flattened between glass slides. To prevent over-flattening of the neocortical tissue, 1.5-mm-thick copper-coated zinc metal spacers (United States Mint) were placed on either side of the cortical tissue. Small weights (∼10 g) were used to apply light pressure to the top glass slide. The duration of flattening was adjusted according to age to account for developmental increases in tissue stiffness and progressive myelination: younger, more pliable tissue (P8) required ∼5 min of light compression, whereas older, more rigid tissue (P20) required up to 30 min to achieve uniform contact against the 1.5 mm metal spacers without damage. This methodology has been reported previously (Dooley and Blumberg, 2018).

Next, the flattened cortex was placed on a platform of frozen PB-sucrose with the cortical surface facing up, and a glass microscope slide was used to apply light pressure to the cortical hemisphere until it was completely frozen. The flattened cortex was then sectioned tangential to the pial surface at 80 µm using a freezing microtome (Leica Microsystems). Following sectioning, wet-mounted tissue was immediately photographed using light and fluorescent microscopy (Leica Microsystems) to identify the relative positioning of fluorescent probes to blood vessels, tissue damage, and any other visual markers.

Sections were then stained for CO, which has been shown in developing rats as young as P5 to reliably delineate primary sensory areas (Seelke et al., 2012). Briefly, cytochrome C (3 mg per 10 ml solution; Sigma-Aldrich), catalase (2 mg per 10 ml solution; Sigma-Aldrich), and 3,3′-diaminobenzidine tetrahydrochloride (DAB; 5 mg per 10 ml solution; Spectrum) were dissolved in a 1:1 dilution of phosphate buffer (PB) and distilled water. Sections were developed in well plates on a rocker at 35–40°C for 3–6 h, after which they were rinsed and mounted. Stained sections were again photographed at 2.5× or 5× magnification, and these individual images were combined into a single composite image.

### Reconstructions

CO staining darkly labels layers IV and VI, with the boundaries of primary cortical areas, particularly the barrels of primary somatosensory cortex (S1) being most striking in layer IV. Thus, our goal with flattening the tissue was to contain layer IV in a single tangential section; however, this was often not possible across the entire neocortical hemisphere. Instead, individual sections often contained only partial cortical areal boundaries (Fig. 2*E*,*F*). However, by photographing the entire series of sections and digitally overlaying them on top of one another (Fig. 2*G*), sequential sections can be combined into a single comprehensive reconstruction (Fig. 2*H*). For each section, the blood vessels, tissue artifacts, fluorescent probes, and architectonic boundaries were used to produce one composite reconstruction. At the lateral portions of the cortex, fluorescent probes begin to resemble lines, rather than distinct points, due to the extreme curvature as the neocortex approaches 90°. When this occurred, the dot reflecting this point on the grid was placed in the most medial location, and the extent of the line of the probe was assumed to be at the same rostral-caudal and medial-lateral position. Using surgical notes, each fluorescent probe was assigned *x*- and *y*-coordinates (relative to bregma).

### Grid straightening

Because flattening the neocortex deforms the tissue—similar to the deformations inherent in transforming the spherical earth onto a flat map—the composite reconstructions of CO-labeled boundaries were digitally corrected using the free-transform tool in Adobe Photoshop (Adobe). Images were systematically warped until the reconstructed grid of fluorescent probe marks was restored to an equidistant stereotaxic matrix (Fig. 2*I*).

### Angle tangential to the cortical surface

Because we flattened the neocortex, we were unable to use our tissue to determine the angle of the cortical surface. But this is often an important detail to consider when performing stereotaxic injections, particularly if the objective is to record perpendicular to the cortical surface. Therefore, we used an existing atlas of developing rat brains, sectioned in the coronal plane, to reconstruct the angle of the neocortex at each point of the grid (Khazipov et al., 2015). These numbers reflected the average of the angle tangential to the cortical surface of the left and right hemispheres for the nearest aged atlas (P8 = P8, P12 = P10, P16 = P14, P20 = P21). While this atlas provides top-down (*x*, *y*) coordinates relative to bregma, stereotaxic dorsal-ventral (DV) coordinates of the cortical surface were not recorded at each insertion site. Because the neocortex is deliberately flattened, physical section thickness postprocessing does not reflect DV depth. When absolute DV surface coordinates are needed, coronal stereotaxic developmental atlases (Khazipov et al., 2015) can be used to determine native surface heights at corresponding AP and ML positions.

### Quantification of S1 barrel coordinates

Stereotaxic coordinates for individual barrels within the S1 barrel field were extracted from high-resolution reconstructions of Long–Evans rats at P8, P12, P16, and P20 using MATLAB (MathWorks). All stereotaxic grid marks (relative to bregma) were registered, allowing us to convert each barrel's coordinates into stereotaxic space. The two-dimensional geometric centroid of each segmented barrel was computed using the regionprops() function and then transformed into stereotaxic space using the fitgeotrans() function to obtain rostral-caudal and medial-lateral coordinates in millimeters relative to bregma for all core barrels (rows A–E) and straddlers (*α*–*δ*; Table 2).

**Table 2.** Stereotaxic coordinates of S1 barrel centroids across early development in Long–Evans rats

Barrel ID	P8		P12		P16		P20	
	RC	ML	RC	ML	RC	ML	RC	ML
E1	−1.36	3.15	−1.96	3.43	−1.74	4.16	−1.69	3.87
E2	−1.00	3.32	−1.59	3.66	−1.38	4.37	−1.23	3.99
E3	−0.69	3.52	−1.31	3.91	−1.02	4.67	−0.81	4.20
E4	−0.48	3.73	−1.12	4.17	−0.70	4.87	−0.66	4.44
E5	−0.39	3.93	−0.92	4.39	−0.61	5.12	−0.46	4.66
E6	−0.27	4.08	−0.89	4.59	−0.52	5.31	−0.35	4.86
E7	−0.35	4.23	−0.96	4.79	−0.50	5.53	−0.33	5.03
D1	−1.70	3.22	−2.41	3.57	−2.18	4.41	−2.15	4.06
D2	−1.35	3.39	−2.08	3.74	−1.87	4.59	−1.67	4.16
D3	−1.14	3.59	−1.83	4.00	−1.51	4.79	−1.34	4.31
D4	−0.87	3.75	−1.54	4.19	−1.26	5.02	−1.13	4.54
D5	−0.77	3.94	−1.41	4.45	−1.02	5.23	−0.94	4.75
D6	−0.67	4.09	−1.39	4.70	−0.92	5.50	−0.83	4.94
C1	−1.96	3.35	−2.82	3.78	−2.44	4.72	−2.53	4.19
C2	−1.73	3.51	−2.51	3.93	−2.16	4.86	−2.10	4.39
C3	−1.43	3.71	−2.17	4.17	−1.87	5.13	−1.82	4.57
C4	−1.14	3.93	−1.89	4.41	−1.54	5.33	−1.51	4.80
C5	−1.00	4.12	−1.72	4.66	−1.28	5.51	−1.25	4.98
B1	−2.20	3.51	−2.96	4.08	−2.70	5.07	−2.73	4.40
B2	−1.96	3.68	−2.74	4.24	−2.46	5.20	−2.49	4.63
B3	−1.75	3.83	−2.45	4.37	−2.15	5.31	−2.18	4.79
B4	−1.53	3.97	−2.23	4.56	−1.89	5.51	−1.89	4.91
A1	−2.45	3.62	−3.17	4.47	−2.94	5.47	−3.01	4.53
A2	−2.40	3.79	−2.89	4.63	−2.66	5.55	−2.87	4.83
A3	−2.10	3.88	−2.65	4.68	−2.40	5.63	−2.63	4.99
A4	−1.81	4.05	−2.45	4.73	−2.16	5.72	−2.37	5.07
α	−2.62	3.43	−3.36	4.21	−3.04	5.22	−3.31	4.50
β	−2.40	3.24	−3.23	3.93	−2.79	4.87	−3.02	4.19
γ	−2.12	3.16	−2.81	3.49	−2.60	4.46	−2.64	3.93
δ	−1.80	3.05	−2.54	3.30	−2.22	4.15	−2.13	3.83

Rostral-caudal (RC) and medial-lateral (ML) centroid coordinates are reported in millimeters relative to bregma (0, 0) for each identifiable macro-barrel across rows A–E and the straddler barrels (*α*, *β*, *γ*, *δ*) at P8, P12, P16, and P20. Negative RC values indicate positions caudal to bregma.

## Results

We reconstructed stereotaxic coordinates over primary cortical areas (S1, A1, and V1) in flattened neocortical tissue for both Sprague Dawley and Long–Evans rat pups at P8, P12, P16, and P20. Across all ages, DiI-labeled stereotaxic probe tracks were visible in tangential sections spanning the entire cortical depth. CO staining reliably labeled layer IV of primary sensory areas, typically spanning two to three adjacent slices (Fig. 2*E*), enabling us to draw precise areal boundaries. Areal boundaries and fluorescent probes were readily aligned, creating comprehensive reconstructions of the developing rat neocortex.

At each age, we observed clear, age-dependent shifts in the relative location of S1, A1, and V1. While the general topography remained consistent—S1 lateral and rostral to V1, A1 lateral and caudal to S1—the absolute position of each area relative to bregma shifted with age. Notably, neocortical expansion from P8 to P20 was nonisometric, expanding preferentially along the rostral-caudal axis compared with the medial-lateral axis (Fig. 1*C*,*D*). In Sprague Dawley rats, the mean rostral-caudal extent of the primary sensory map increased from 8 mm at P8 to 11 mm at P20 (a 38% increase; 3 mm total growth), whereas medial-lateral extent increased from 5 mm to 6 mm (a 20% increase; 1 mm total growth). A similar bias occurred in Long–Evans rats, where rostral-caudal span expanded from 9 mm at P8 to 11.5 mm at P20 (a 28% increase; 2.5 mm total growth), compared with an increase in medial-lateral span from 5.5 to 6 mm (a 9% increase; 0.5 mm total growth). Across both strains, the relative growth along the rostral-caudal axis outpaced medial-lateral expansion by over twofold. These distortions underscore the limitations of simply scaling adult stereotaxic coordinates down to pup brains.

After aligning CO-stained maps with stereotaxic probe locations, we generated warped, age-specific top-down reconstructions for Long–Evans rats (Fig. 3) and Sprague Dawley rats (Fig. 4). These maps provide stereotaxic coordinates for primary cortical areas from P8 to P20. Independent reconstructions from the two animals at each age exhibited closely matched structural profiles, confirming that the spatial relationships between primary cortical areas and grid coordinates were conserved. Minor strain differences were observed: Long–Evans rats showed slightly larger and more caudally shifted sensory areas compared with Sprague Dawley rats at matched ages, likely reflecting differences in skull and brain morphology.

**Figure 3.**
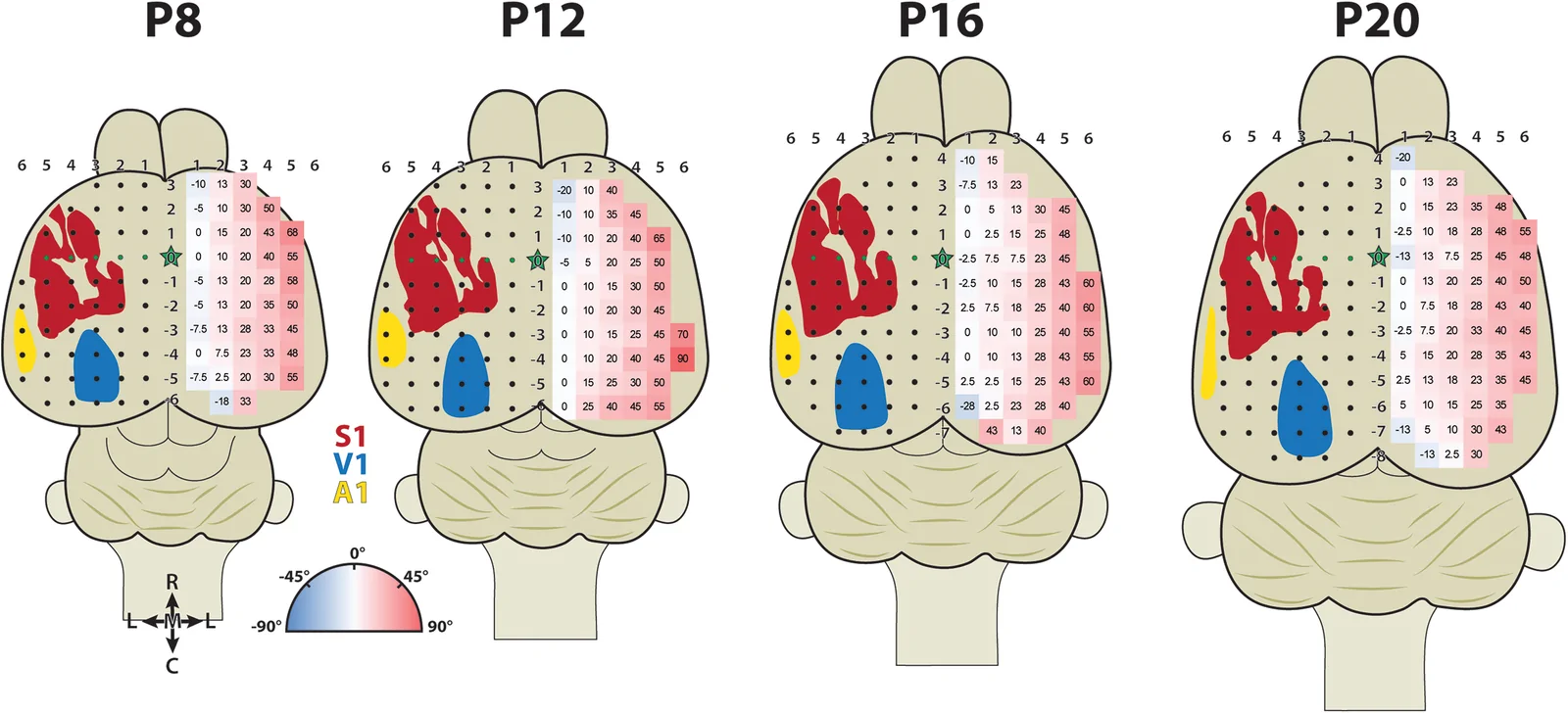
Developmental stereotaxic coordinates and neocortical surface angles in Long–Evans rats. Top-down reconstructions of the neocortex at P8, P12, P16, and P20. On the left neocortical hemisphere, the boundaries of primary sensory areas derived from flattened CO-stained tissue are shown, including the primary somatosensory cortex (S1, red), primary visual cortex (V1, blue), and primary auditory cortex (A1, yellow). Black dots indicate stereotaxic coordinate target locations of the 1 mm grid across the hemisphere, with the green-filled dots indicating bregma (0 mm AP). The green star indicates the location of bregma on the midline. On the right, the local angle of the neocortical surface relative to the horizontal plane (0°) measured at each grid intersection point, taken from Khazipov et al. (2015) (see Materials and Methods for more details). Numbers inside the grid are the angle tangential to the cortical surface in degrees. Red shading corresponds to positive angles (up to 90°), blue shading corresponds to negative angles (down to −90°), and white indicates a horizontal plane. Medial-lateral grid columns (top numbers) and rostral-caudal grid rows (midline numbers) indicate absolute coordinates in millimeters relative to bregma (0, 0).

**Figure 4.**
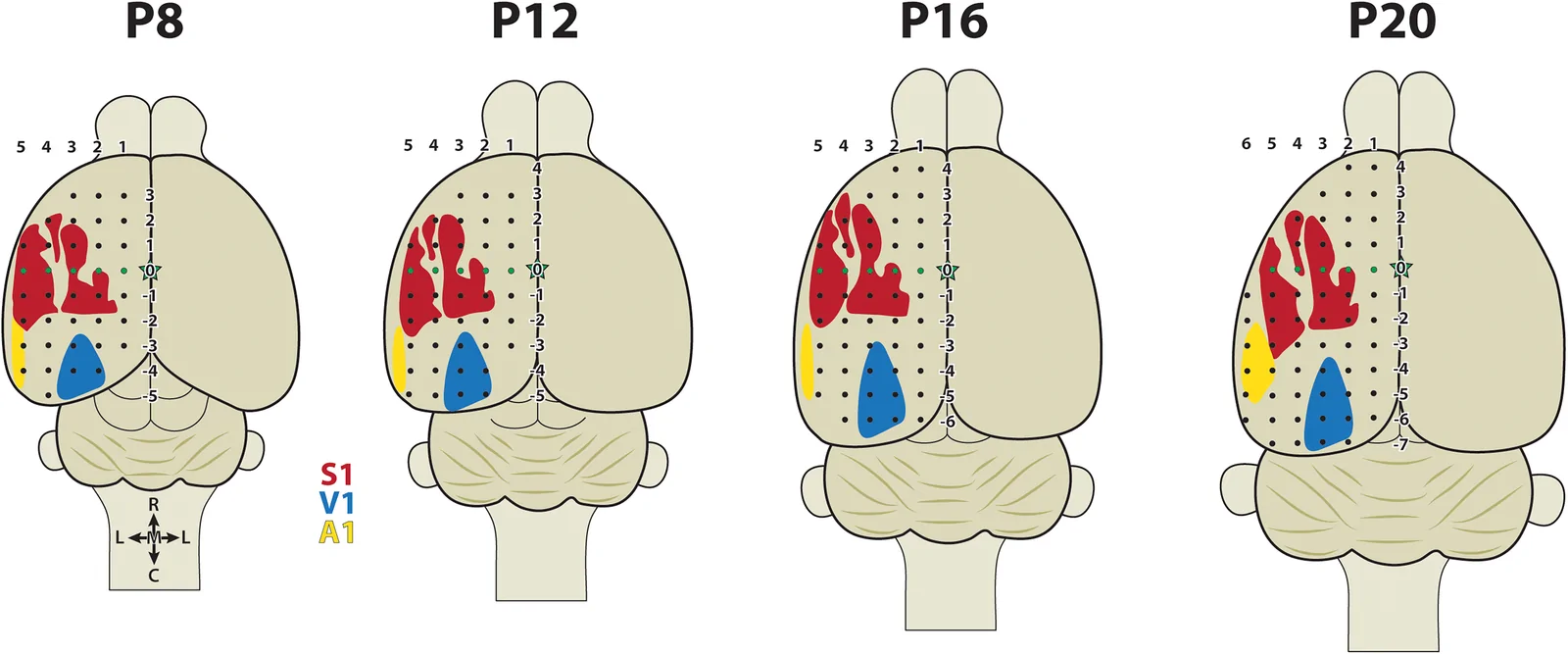
Developmental stereotaxic coordinates in Sprague Dawley rats. Top-down reconstructions of primary sensory areas (S1, V1, and A1) mapped relative to the 1 mm stereotaxic grid at P8, P12, P16, and P20. All layout and labeling conventions as in Figure 3, with the exception of the neocortical surface angle maps.

To demonstrate how the neocortical grids aligned onto localized functional subdivisions, we generated high-magnification reconstructions of the S1 barrel field in Long–Evans rats across all four ages (Fig. 5). Tangential CO staining clearly resolved individual barrels (rows A1–E1) at P8, with the structural definition of distinct barrel walls distinctly visible at all ages (Fig. 5, left panels). Anatomical schematics illustrate the representative spatial relationships between these functional modules and nearby stereotaxic coordinate intersections (Fig. 5, right panels). Centroid stereotaxic coordinates (rostral-caudal and medial-lateral relative to bregma) for all identifiable barrels and straddlers across each developmental age are provided in Table 2. Reflecting the nonlinear expansion observed across the hemisphere, the position of the barrel field relative to fixed stereotaxic coordinates shifts systematically across development. For example, the (−1, 4) coordinate target sits near the rostro-medial border of the barrel field at P8 but occupies an internal position near the E1/D1 rows by P16, capturing a local outward and forward displacement of the somatosensory map relative to skull landmarks. Importantly, while the precise boundaries of individual barrels exhibit expected biological variability, the overall topographic progression follows a conserved developmental trajectory, providing a representative reference framework for targeting functional columns within S1.

**Figure 5.**
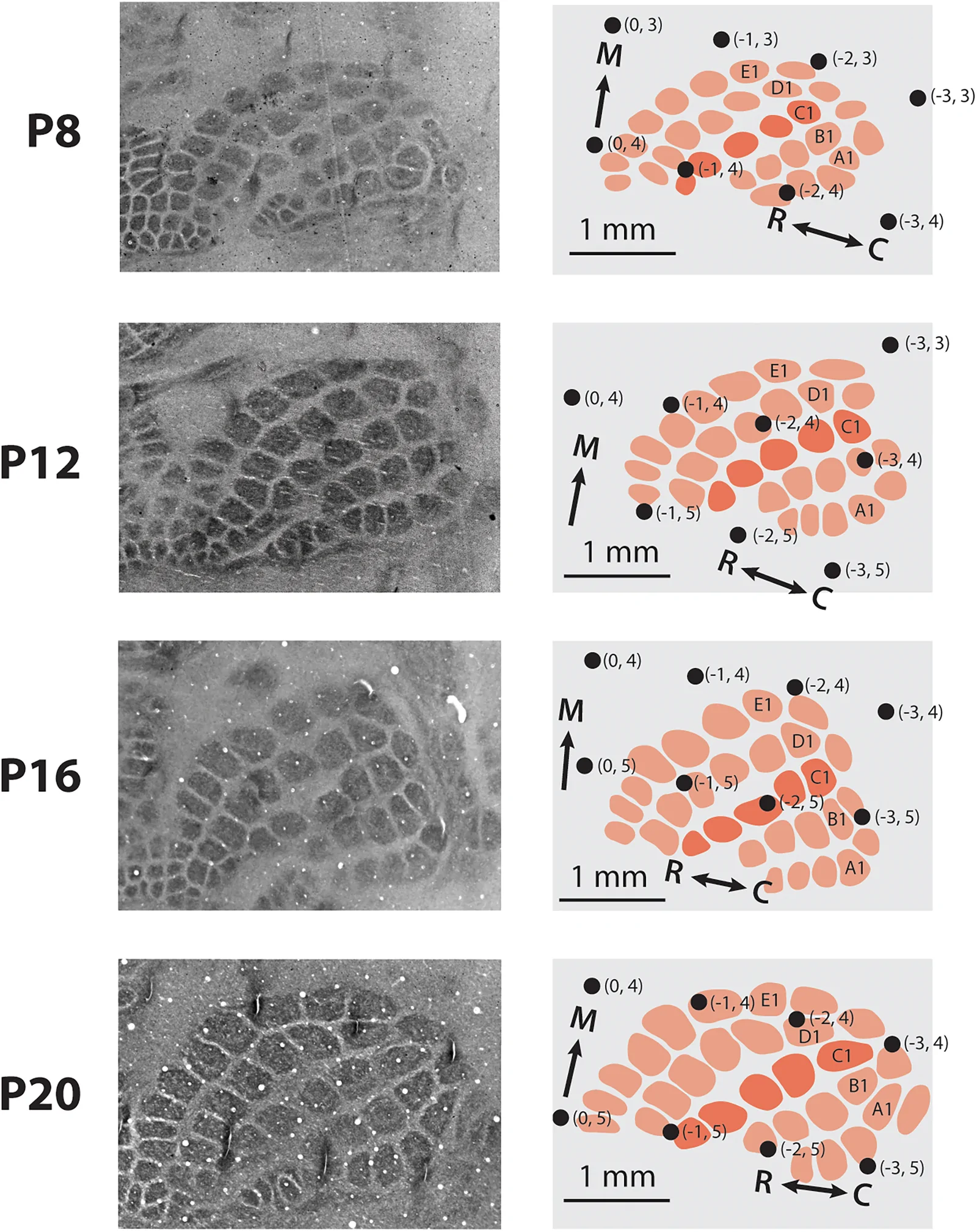
High-magnification developmental tracking of the Long–Evans rat S1 barrel field. Reconstructions track the location of the barrel subfield across four early postnatal ages: P8, P12, P16, and P20. Left, Photomicrographs of tangential neocortical sections processed for CO staining, showing the distinct walls of individual barrels. Right, Corresponding anatomical illustrations mapping the layout of barrels (red ovals), labeling the first whisker of all rows (A–E). Row C is shown in darker red than other rows. Black dots indicate stereotaxic coordinates with absolute values given in millimeters relative to bregma (R-C, M-L). Scale bars, 1 mm.

## Discussion

The present atlas provides a resource for rat researchers in developmental systems neuroscience by replacing resource-intensive, laboratory-specific trial-and-error coordinate mapping with a standardized, anatomically verified atlas of primary cortical areas. Our results confirm that the growth of the infant rat neocortex from P8 to P20 does not follow a simple, isometric scaling law (Bandeira et al., 2009; Khazipov et al., 2015). Specifically, cortical expansion between P8 and P20 is biased along the rostral-caudal axis, which expanded by 28–38% across strains compared with only 9–20% along the medial-lateral axis. By capturing these regional growth distortions and mapping them relative to functional structural boundaries—such as the S1 vibrissae macro-barrels—this atlas provides a reliable blueprint for targeted neurophysiological recordings or viral delivery approaches.

The current age range includes several major functional transitions, including the onset of spatially and temporally precise sensory responses (Colonnese et al., 2010; Dooley and Blumberg, 2018; Dooley et al., 2021; Makarov et al., 2021), behavioral-state-dependent activity (Murata and Colonnese, 2018; Gómez et al., 2021), and eye and ear opening (Colonnese and Phillips, 2018). Importantly, the methods used here are not appropriate at earlier postnatal ages because prior to P5, CO staining fails to resolve distinct areal boundaries (Seelke et al., 2012). However, several developmental atlases exist that focus on the first postnatal week (Khazipov et al., 2015; Ramachandra and Subramanian, 2016) and even prenatal brains (Altman and Bayer, 1995; Paxinos and Ashwell, 2018), relying on different histological techniques to visualize structure within the tissue. And on the other end of development, neocortical and skull growth is not entirely complete at P20; bregma and lambda landmarks have not yet reached adult-like distances in either strain by this stage (Fig. 1*B*). However, beyond P20, the extreme nonlinear growth dynamics characteristic of early postnatal development begins to stabilize. Consequently, standard coordinate scaling based on adult bregma–lambda distances is more likely to yield accurate, predictable stereotaxic results in juvenile rats.

## Synthesis

Reviewing Editor: Rosemary Bagot, McGill University Decisions are customarily a result of the Reviewing Editor and the peer reviewers coming together and discussing their recommendations until a consensus is reached. When revisions are invited, a fact-based synthesis statement explaining their decision and outlining what is needed to prepare a revision will be listed below. The following reviewer(s) agreed to reveal their identity: Rustem Khazipov.

The atlas provides barrel-level precision of the developing sensory cortex in stereotaxic co-ordinates. It is a well-executed and useful resource paper. The authors combine stereotaxic DiI probe mapping with cytochrome oxidase histochemistry on flattened neocortical tissue to generate top-down maps of S1, A1, and V1 across four early postnatal ages (P8-P20) in two commonly used rat strains and pair these with surface-angle data from an existing coronal atlas. The dataset fills a genuine methodological gap as most existing developmental atlases rely on coronal sections that obscure areal topography, and should be of direct practical value to labs performing stereotaxic surgery in neonatal/juvenile rats.

The following suggestions should be addressed to provide additional methodological details and strengthen the quantitative support for the paper's central claim about non-isometric cortical expansion.

1)Please provide additional details on how the head was fixed in the stereotaxic apparatus, including the measures taken to ensure its consistent spatial positioning. Were bregma and lambda at the same height? Were the heights at symmetric lateral positions similar? It would be informative to include a table presenting the height of each insertion point relative to bregma.

2) The presentation of the barrel map in stereotaxic coordinates is excellent (Fig. 5). However, the authors could extract more useful information from this by providing a detailed table with the coordinates of all identifiable barrels, along with the depth from the cortical surface (at all ages and in both rat strains). Since lateral structures are often accessed via angled recordings, this information-combined with cortical curvature data from other atlases-could enable precise positioning of recording electrodes or imaging with barrel-level accuracy.

3) The text states that "the animal with the best histology quality was selected for the atlas," but this criterion is not defined. Since only one of two animals per age/strain group is carried forward into the atlas, readers evaluating reproducibility need to know what "best" means here - e.g., tissue flatness, staining contrast, absence of tears/folds, clarity of layer IV boundaries. An operational definition (even a brief one) would help readers judge how the selection was made and whether it could introduce bias.

4) The Methods state that Sprague-Dawley and Long-Evans rats were used but don't list a vendor/breeding source. Please add this - strain substrains can differ meaningfully in developmental timing and morphology, so vendor information is relevant to reproducibility.

5) The tissue was "flattened between glass slides for 5-30 minutes," which is a fairly wide range. Please specify what determined where a given sample fell within that range (e.g., age, tissue thickness, visual assessment of flatness) so other labs can standardize their own protocol.

6) Quantitative support for the non-isometric expansion claim (Results, line ~188-189; Discussion, line ~219-220) is lacking. This is arguably the paper's key structural finding: that neocortical growth from P8-P20 is biased toward the rostral-caudal axis over the medial-lateral axis. However, it is stated qualitatively in both the Results and Discussion. This is an important claim that would be considerably strengthened by an actual numerical measure (e.g., fold-change or mm expansion along each axis, with some measure of variability across animals) rather than "increased much more than." As written, it is hard for a reader to judge the magnitude or reliability of the effect.

Minor comments:

• Consider double-checking that pairing of specific vendor/reagent details is consistent throughout Methods (some reagents list a supplier, e.g., DiI, DAB; others, like the animals themselves, do not).

## Author Response

Response to Reviewer Comments We thank the reviewers and the editor for their construc ve feedback and posi ve evalua on of our manuscript. We have addressed all sugges ons by adding quan ta ve growth data, detailing surgical posi oning, providing an opera onal defini on of histological selec on criteria, and genera ng a dedicated reference table of stereotaxic coordinates for individual S1 barrels across early development. In the manuscript, all the revised text is highlighted in light gray. Below is our point-by-point response detailing the revisions.

Comment 1: Please provide addi onal details on how the head was fixed in the stereotaxic apparatus, including the measures taken to ensure its consistent spa al posi oning. Were bregma and lambda at the same height? Were the heights at symmetric lateral posi ons similar? It would be informa ve to include a table presen ng the height of each inser on point rela ve to bregma.

Response: We apologize for this omission in our original dra and have updated the Methods sec on (Surgery) to detail head fixa on and flat-skull leveling procedures. Briefly, rat pups were secured in a stereotaxic apparatus using non-puncture ear bars together with a custom nose cone (P8, P12) or incisor bar (P16, P20). Flat-skull alignment was verified across two orthogonal planes using a stereotaxic microprobe: rostral-caudal lt was adjusted un l the dorsal-ventral (DV) coordinate difference between bregma and lambda was < 0.05 mm, and medial-lateral level was verified by confirming that DV coordinates at symmetric posi ons roughly midway between bregma and lambda ({plus minus} 2 mm lateral to the sagi al suture) also differed by < 0.05 mm.

Regarding a table of inser on heights rela ve to bregma: because our experimental approach relies on physically fla ening the cor cal hemisphere between glass slides to visualize tangen al areal boundaries, physical sec on thickness post-processing does not preserve na ve, in vivo DV surface curvature. Further, we did not log the DV coordinate during probe inser ons. To guide users who require absolute stereotaxic DV coordinates, we have added an explicit statement in the Methods direc ng researchers to published high-density coronal atlases (e.g., Khazipov et al., 2015) to obtain na ve surface heights at corresponding RC and ML posi ons.

Comment 2: The presenta on of the barrel map in stereotaxic coordinates is excellent (Fig. 5). However, the authors could extract more useful informa on from this by providing a detailed table with the coordinates of all iden fiable barrels, along with the depth from the cor cal surface (at all ages and in both rat strains). Since lateral structures are o en accessed via angled recordings, this informa on combined with cor cal curvature data from other atlases-could enable precise posi oning of recording electrodes or imaging with barrel-level accuracy.

Response: We agree that an explicit coordinate table makes this resource significantly more prac cal for targeted electrophysiological and op cal recordings. We have added Table 2, which provides the exact rostral-caudal and medial-lateral stereotaxic centroid coordinates rela ve to bregma for all 30 core barrels (rows A-E) and straddler barrels across development (P8, P12, P16, and P20). We also added a subsec on to the Methods (Quan fica on of S1 Barrel Coordinates) describing how we used MATLAB to segment barrels from the image and convert them to stereotaxic coordinates.

Because high-magnifica on reconstruc ons of individual barrel walls were generated exclusively in Long Evans rats (Figure 5), Table 2 provides coordinate data for this strain.

Comment 3: The text states that "the animal with the best histology quality was selected for the atlas," but this criterion is not defined. Since only one of two animals per age/strain group is carried forward into the atlas, readers evalua ng reproducibility need to know what "best" means here - e.g., ssue flatness, 1 staining contrast, absence of tears/folds, clarity of layer IV boundaries. An opera onal defini on (even a brief one) would help readers judge how the selec on was made and whether it could introduce bias.

Response: We have updated the Methods sec on (Paragraph 1) to provide an opera onal defini on of histological quality: "For each age and strain, the animal with the highest histological quality was selected for inclusion in the atlas. Histological quality was opera onally defined by two criteria: (1) opmal tangen al sec oning planarity, such that layer IV across primary sensory areas was captured across the minimum number of sequen al sec ons (typically 2-3), and (2) high contrast and clear laminar/areal differen a on of CO staining, allowing unambiguous iden fica on of primary sensory borders and individual barrel walls." Comment 4: The Methods state that Sprague-Dawley and Long-Evans rats were used but don't list a vendor/breeding source. Please add this - strain substrains can differ meaningfully in developmental ming and morphology, so vendor informa on is relevant to reproducibility.

Response: We have added commercial breeder details to the Methods sec on. Long-Evans breeders were obtained from Envigo (now Ino v, Indianapolis, IN) and Sprague-Dawley breeders were acquired from Charles River Laboratories (Wilmington, MA). All experimental animals were bred, born, and reared in house under iden cal environmental condi ons.

Comment 5: The ssue was "fla ened between glass slides for 5-30 minutes," which is a fairly wide range.

Please specify what determined where a given sample fell within that range (e.g., age, ssue thickness, visual assessment of flatness) so other labs can standardize their own protocol.

Response: We have clarified in the Methods (Histology) that compression dura on scaled directly with developmental age to accommodate age-dependent increases in ssue s ffness and progressive myelina on. P8 ssue is so and pliable, requiring only ~5 minutes of compression, whereas more rigid P20 ssue required up to 30 minutes to achieve uniform contact against the 1.5 mm spacers without ssue fracture or damage.

Comment 6: Quan ta ve support for the non-isometric expansion claim (Results, line ~188-189;

Discussion, line ~219-220) is lacking. This is arguably the paper's key structural finding: that neocor cal growth from P8-P20 is biased toward the rostral-caudal axis over the medial-lateral axis. However, it is stated qualita vely in both the Results and Discussion. This is an important claim that would be considerably strengthened by an actual numerical measure (e.g., fold-change or mm expansion along each axis, with some measure of variability across animals) rather than "increased much more than." As wri en, it is hard for a reader to judge the magnitude or reliability of the effect.

Response: We have quan fied cor cal expansion across development and added these data as panels C and D to Figure 1. In addi on, we added numerical expansion values (both absolute millimeters and percentage increase normalized to P8) to the Results and Discussion.

In Sprague-Dawley rats, the rostral-caudal span of the primary sensory map grew from 8.0 mm to 11.0 mm (+38%; 3.0 mm total growth), whereas the medial-lateral span grew from 5.0 mm to 6.0 mm (+20%;

1.0 mm total growth).

In Long-Evans rats, the rostral-caudal span expanded from 9.0 mm to 11.5 mm (+28%; 2.5 mm total growth), compared to an increase in medial-lateral span from 5.5 mm to 6.0 mm (+9%; 0.5 mm total growth).

2 Across both strains, rela ve elonga on along the rostral-caudal axis outpaced medial-lateral expansion by more than two-fold.

Minor Comments: Consider double-checking that pairing of specific vendor/reagent details is consistent throughout Methods (some reagents list a supplier, e.g., DiI, DAB; others, like the animals themselves, do not).

Response: We reviewed the en re Methods sec on and verified that all equipment, so ware, and chemical reagents include supplier names and loca ons on first men on (e.g., Stoel ng Co., Covetrus, Sigma-Aldrich, Leica Microsystems, Adobe Inc., and MathWorks).
